# Impact of the seventh day nucleated red blood cell count on mortality in COVID-19 intensive care unit patients: A retrospective case-control study

**DOI:** 10.5937/jomb0-39839

**Published:** 2023-01-20

**Authors:** Muhammed Emin Düz, Mustafa Arslan, Elif E. Menek, Burak Yasin Avci

**Affiliations:** 1 Amasya University, Sabuncuoğlu Şerefeddin Training, and Research Hospital, Medical Biochemistry, Amasya, Turkey; 2 Amasya University, Sabuncuoğlu Şerefeddin Training, and Research Hospital, Infectious Diseases, Amasya, Turkey

**Keywords:** nucleated erythrocytes, COVID-19, COVID19 pandemic, mortality, survival, nukleirani eritrociti, COVID-19, COVID19 pandemija, mortalitet, preživljavanje

## Abstract

**Background:**

COVID-19 covers a broad clinical spectrum, threatening global health. Although several studies have investigated various prognostic biochemical and hematological parameters, they generally lack specificity and are insufficient for decision-making. Beyond the neonatal period, NRBCs (nucleated red blood cells) in peripheral blood is rare and often associated with malignant neoplasms, bone marrow diseases, and other severe disorders such as sepsis and hypoxia. Therefore, we investigated if NRBCs can predict mortality in hypoxic ICU (Intensive Care Unit) patients of COVID-19.

**Methods:**

Seventy-one unvaccinated RT-PCR confirmed COVID-19 ICU patients was divided into those who survived (n=35, mean age=58) and died (n=36, mean age=75). Venous blood samples were collected in K3 EDTA tubes and analyzed on a Sysmex XN-1000 hematology analyzer with semiconductor laser flow cytometry and nucleic acid fluorescence staining method for NRBC analysis. NRBC numbers and percentages of the patients were compared on the first and seventh days of admission to the ICU. Results are reported as a proportion of NRBCs per 100 WBCs NRBCs/100 WBC (NRBC% and as absolute NRBC count (NRBC #, × 109/L).

**Results:**

NRBC 7th-day count and % values were statistically higher in non-survival ones. The sensitivity for 7th day NRBC value <0.01 (negative) was 86.11%, the specificity was 48.57%, for <0.02; 75.00%, and 77.14%, for <0.03; 61.11%, and 94.60%.

**Conclusions:**

In conclusion, our results indicate that NRBC elevation (>0.01) significantly predicts mortality in ICU hospitalized patients due to COVID-19. Worse, a high mortality rate is expected, especially with NRBC values of >0.03.

## Abstract (sr)

**Uvod:**

COVID-19 pokriva širok klinički spektar, ugrožavajući globalno zdravlje. Iako je nekoliko studija istraživalo različite prognostičke biohemijske i hematološke parametre, njima generalno nedostaje specifičnost i nedovoljni su za donošenje odluka. Nakon neonatalnog perioda, NRBC (crvena krvna zrnca sa jezgrom) u perifernoj krvi su retka i često povezana sa malignim neoplazmama, bolestima koštane srži i drugim teškim poremećajima kao što su sepsa i hipoksija. Stoga smo istražili da li NRBC mogu predvideti smrtnost kod hipoksičnih pacijenata na intenzivnoj nezi (intenzivne nege) pacijenata sa COVID-19.

**Metode:**

Sedamdeset i jedan nevakcinisani RT-PCR pacijent koji je potvrdio COVID-19 ICU je podeljen na one koji su preživeli (n=35, prosečna starost=58) i umrli (n=36, prosečna starost=75). Uzorci venske krvi su sakupljeni u K3EDTA epruvete i analizirani na Sysmex KSN-1000 hematološkom analizatoru sa poluprovodničkom laserskom protočnom citometrijom i metodom fluorescentnog bojenja nukleinskom kiselinom za analizu NRBC. Broj i procenat NRBC pacijenata su upoređeni prvog i sedmog dana prijema u intenzivnoj nezi. Rezultati su prikazani kao proporcija NRBC na 100 belih krvnih zrnaca [NRBC/100 belih krvnih zrnaca (NRBC%] i kao apsolutni broj NRBC (NRBC # , x 109/L).

**Rezultati:**

Broj NRBC 7. dana i vrednosti u procentima bili su statistički veći kod onih koji nisu preživeli. Osetljivost za NRBC 7. dana vrednost <0,01 (negativna) bila je 86,11%, specifičnost 48,57%, za <0,02; 75,00% i 77,14% za <0,03; 61,11% i 94,60%.

**Zaključak:**

U zaključku, nasi rezultati pokazuju da povišenje NRBC (> 0,01) značajno predviđa smrtnost kod pacijenata koji su hospitalizovani na intenzivnoj nezi zbog COVID-19. Sto je još gore, očekuje se visoka stopa mortaliteta, posebno sa vrednostima NRBC > 0,03.

## Introduction

COVID-19 covers a broad clinical spectrum, from asymptomatic to severe pneumonia with multiorgan failure, threatening global health [1]. Early diagnosis of critically ill patients can reduce mortality with timely interventions [2]. Several studies have investigated various factors’ diagnostic or prognostic value, including age, sex, CT scan, biochemical and hematological parameters [3] [4] [5] [6] [7] [8]. Although changes in these parameters are highly suggestive of the severity of the disease and mortality, their specificity is low and may not be sufficient for decision-making. The parameters that predict disease course are widely available, but their values are limited by significant inter-patient variability and specificity. At this point, the need for a parameter that can predict mortality, especially in inpatients and intensive care unit (ICU) patients, is vital.

‘NRBC’ – the term ‘nucleated red blood cells’ refers to progenitor cells of the red blood cell lineage that still contain a nucleus; they are also known as erythroblasts or – old – normoblasts. NRBC can only be found in the blood-forming bone marrow, where they mature in healthy adults and older children. Their appearance in peripheral blood indicates extramedullary erythropoiesis (outside the bone marrow) or disruption of the blood-bone marrow barrier. Both possible scenarios can only be found in the course of severe disease. Physiologically, NRBC occurs only in the peripheral blood of neonates and premature infants [9]. Beyond the neonatal period, NRBCs in peripheral blood are rare and often associated with malignant neoplasms, bone marrow diseases, and other serious disorders such as sepsis and hypoxia [10] [11] [12].

For COVID-19 ICU patients, the turning point for a worse prognosis appears to be the onset of progressive hypoxia, exacerbated by a poorly understood host response involving a cytokine storm [13]. At this point, cytokine storms and severe hypoxia are thought to be the leading causes of death. Therefore, NRBCs can predict mortality in ARDS with high predictive power in ICU patients caused by COVID-19, similar to other diseases. In addition, the presence of NRBCs in the blood can be considered a marker of disease severity, indicating a higher risk of ICU death [14]. In this context, mortality can be predicted in COVID-19 patients using NRBC values, and treatment could be possible at a key point in the clinical course. Therefore this study aims to examine the predictive potential of NRBC for mortality in COVID-19 patients. We believe our trial data will yield discoveries that will help minimize mortality in these individuals.

## Materials and methods

### Study population

Seventy-one unvaccinated RT-PCR confirmed COVID-19 ICU patients (47 males and 24 females, mean age 65, range 22–93) were included in the study group between April 2020 and June 2020. The patient group was divided into those who survived (n=35, mean age=58) and died (n=36, mean age=75). The patients were further subdivided into subgroups according to total comorbidities for COVID-19 as a whole, such as diabetes, coronary artery disease, hypertension, obesity, and pregnancy. Of 71 COVID-19 ICU patients, 25 had diabetes mellitus (DM), 35 had coronary artery disease (CAD), 43 had hypertension (HT), 19 had obesity, and 3 had a pregnancy. A study cohort of 404 ICU patients demonstrated that peak NRBC values were achieved on day 7 [14]. Based on this study, we examined the NRBC values of our patients on the seventh day and the day of hospitalization in the ICU [14]. Therefore, NRBC numbers and percentages of the patients were recorded on the first and seventh days of admission to the ICU.

After obtaining a COVID-19 work permit and IRB confirmation from the Ministry of Health of Turkey, the study was initiated after receiving ethics committee approval from the Amasya University Faculty of Medicine.

### Methods

A venous blood sample was collected in tubes with standard complete blood count anticoagulant K3 EDTA (Vacutainer® Beckton-Dickinson) and analyzed on Sysmex XN-1000 (Sysmex Corporation, Kobe, Japan) hematology analyzers within 4 hours of collection.

The Sysmex XN-1000 analyzer makes an accurate NRBC analysis with an excellent correlation to the microscope count and a flow cytometry reference method. A specific reagent (fluorochrome-polymethine dye) completely lyses the red blood cell (RBC), enucleates, shrinks, and lightly stains the nuclei of the NRBC. The lysing reagent preserves the shape of the white blood cell (WBC) while intensely staining its cytoplasmic organelles and nuclei. These different staining intensities and their different volumes between NRBC cores and WBCs are detected by a semiconductor laser using forward-scattered light and fluorescent intensities. As a result, a clear separation of the two cell populations occurs. Results are reported as a proportion of NRBCs per 100 WBCs (NRBCs/100 WBC (NRBC%) and as absolute NRBC count NRBC #, × 10^9^/L). Information about the working methodology of the device has been obtained from the manufacturer, and detailed information can be obtained from the website. (https://www.sysmex.co.jp/en/products_solutions/library/journal/vol30_no1/summary01/vol30_1_01.pdf).

### Statistical analysis

Analyzes were performed using SPSS for Windows, Version 24 (SPSS Inc., Chicago, USA), and Microsoft Excel (Microsoft, Washington, USA). The Shapiro-Wilk test confirmed the distribution normality of the data before further analysis. Descriptive statistics are evaluated as the mean, standard error of the mean, median (Interquartile range, IQR), minimum and maximum. Because variables are parametric, intergroup comparisons of clinical and hematological parameters were evaluated with an Independent samples t-test. Receiver operating characteristics (ROC) and relevant area-under-curve (AUC) analyses were performed for parameters to differentiate the COVID-19 prognosis. Binary Logistic regression via multivariate analysis was used to exploit risk factors for mortality. The level of significance was considered at 0.05.

## Results

No difference was observed between NRBC values regarding mortality between genders (p=0.056). However, the age of the patients who died was significantly higher, as expected. Although diabetes mellitus, obesity, and pregnancy did not have a significant effect on mortality (p=0.189, p=0.847, p=0.578, respectively), there was a substantial effect on coronary artery disease (CAD) and hypertension (HT) (p<0.001, and p=0.001, respectively). Thus, when the comorbidity was considered a total, it harmed survival. However, the relationship between individual or total comorbidity factors and NRBC values could not be determined. Descriptive statistics according to mortality are given in Table 1.

**Table 1:** Descriptive statistics according to survival (0) and mortality (1). SE: Standard error of the mean, <br>IQR: Interquartile range, <br>NA: Not applicable. <br>NRBC: Nucleated Red Blood Cell.

Descriptive Statistics								
		Median	Mean	SE	IQR	Minimum	Maximum	p-value
NRBC 1st Day	0	0.000	0.000	0.000	0.000	0.000	0.000	NA
NRBC 1st Day	1	0.000	0.002	0.001	0.000	0.000	0.040	
NRBC% 1st Day	0	0.000	0.000	0.000	0.000	0.000	0.000	NA
NRBC% 1st Day	1	0.000	0.019	0.010	0.000	0.000	0.200	
NRBC 7th Day	0	0.010	0.010	0.003	0.010	0.000	0.100	0.001
NRBC 7th Day	1	0.040	0.131	0.034	0.142	0.000	0.800	
NRBC% 7th Day	0	0.010	0.083	0.020	0.100	0.000	0.500	<0.001
NRBC% 7th Day	1	0.250	0.543	0.118	0.575	0.000	3.400	
Days of Hospitalization	0	18.000	20.371	2.025	14.500	4.000	52.000	0.391
Days of Hospitalization	1	15.000	17.556	2.545	14.500	2.000	89.000	
Age	0	56.000	56.000	2.218	16.000	22.000	79.000	<0.001
Age	1	77.500	75.111	2.435	14.500	26.000	93.000	
Comorbidity	0	1.000	1.400	0.202	2.000	0.000	4.000	0.002
Comorbidity	1	2.000	2.139	0.121	1.000	1.000	4.000	

**Figure 1:**
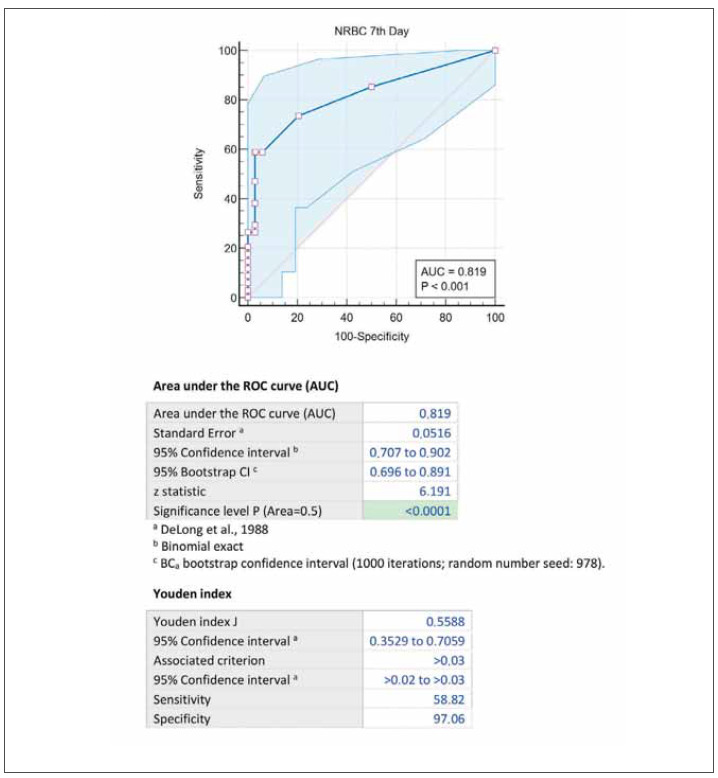
According to survival, the ROC area under curve (AUC) and Youden index analysis of 7th-day NRBC levels.

Since NRBC 1st-day numbers and percentages mostly gave »0« results, analysis on mortality was not applicable. In contrast, NRBC 7th-day count and % values were statistically higher in non-survival ones. There was no difference between survival and nonsurvival groups regarding the length of stay in the ICU. However, the effect of NRBC measurements on mortality was investigated using ROC curves. Figure 1 represents the ROC curve for 7th-day NRBC values on mortality.

To determine the cut-off value on mortality, the test’s sensitivity for NRBC value <0.01 (negative) was 86.11%, the specificity was 48.57%, for <0.02; 75.00%, and 77.14%, for <0.03; 61.11%, and 94.60%. A prognostic evaluation of 7th-day NRBC’s effect on mortality is given in Table 2.

**Table 2:** The prognostic evaluation of 7th-day NRBC's effect on mortality. AUC: Area Under Curve.

	NRBC<0.01<br>(negative)		NRBC<0.02		NRBC<0.03	
Sensitivity	86.11%	70.503% to<br>95.332%	75.00%	57.797% to<br>87.880%	61.11%	43.464% to<br>76.858%
Specificity	48.57%	31.383% to<br>66.011%	77.14%	59.864% to<br>89.579%	94.60%	81.805% to<br>99.339%
AUC	0.673	0.552 to 0.780	0.761	0.645 to 0.854	0.779	0.666 to 0.867
Positive Likelihood Ratio	1.674	1.183 to 2.371	3.281	1.735 to 6.205	11.306	2.864 to 44.621
Negative Likelihood Ratio	0.286	0.118 to 0.691	0.324	0.179 to 0.587	0.411	0.271 to 0.624
Positive Predictive Value	63.27%	54.883% to<br>70.916%	77.14%	64.088% to<br>86.455%	91.67%	73.594% to<br>97.749%
Negative Predictive Value	77.27%	58.464% to<br>89.146%	75.00%	62.358% to<br>84.455%	71.43%	62.237% to<br>79.133%
Accuracy	67.61%	55.453% to<br>78.237%	76.06%	64.455% to<br>85.390%	78.08%	66.862% to<br>86.922%

## Discussion

Our study suggests that negative NRBC results of COVID-19 ICU patients correlate with survival. However, mortality increases with NRBC values of 0.01. NRBC value >0.03 indicates mortality with a specificity of 97.06%. When we take the cut-off value of 0.01 for NRBC, the sensitivity is 86.11%, and the specificity is 48.57%; For 0.03, it was 61.11% and 94.60%. Positive NRBC values mean a warning for early mortality, while high values indicate ‘a perfect storm’ for patients. NRBC% levels rose much greater in deceased patients than in survivors. NRBC% is calculated in proportion to white blood cells (WBC) and is affected by various changes in WBC values. For this reason, we find it more meaningful to consider NRBC measurements as numbers. A study found that 54% of patients admitted to a cardiac ICU were NRBC positive [15]. Some other studies report lower values of 17.5 and 24.8% in septic or surgical patients [11] [12] [16] [17]. Studies conducted in ARDS (Acute respiratory distress syndrome) cases, which can cause inflammation and respiratory distress, and mortality similar to the clinical picture in COVID-19 patients hospitalized in the ICU, have revealed the connection between NRBC increase and mortality similar to our study results [11] [12] [16] [17] [18] [19] [20]. Thus, our study results are compatible with the literature data. In addition, although the association of CAD and HT diseases with COVID-19 increases mortality, we could not detect their relationship with NRBC values.

In a study conducted in COVID-19 patients, compared with survivors, a significant increase has been found in neutrophil percentage, platelet distribution with, platelet-large cell percentage, MPV, NRBC, and NRBC% [21]. In another study, NRBC values were significantly higher in COVID-19 ICU patients than in non-ICU patients [22]. Finally, in a study conducted on patients admitted to the emergency department, NRBC values were slightly higher in COVID-19 positive cases than negative ones. Still, this elevation was not found in hospitalized patients [23]. This can be interpreted as the NRBC values will not increase immediately since day 1 NRBC values did not increase in our study data since systemic inflammation and hypoxia that would increase NRBC values in the patient did not occur in the first days of admission to the hospital.

Thus, although the exact mechanism by which NRBCs are finally released from the bone marrow into circulation remains unclear, their presence in peripheral blood is a valid marker for disease severity and increased mortality. However, arterial hypoxemia with high levels of proinflammatory cytokines and systemic inflammation has been suggested to be potent triggering factors for releasing NRBCs into the peripheral circulation [24] [18] [25] [26]. In addition, researchers have pointed out that NRBCs were identified as a robust predictor of postdischarge mortality in critically ill patients who survived hospitalization [27].

## Limitations

Our study has several limitations. One of them was the low number of our patients. We believe that studies with a more significant number of patient data will yield detailed results. Another is that we could not reveal how much NRBC values would be associated with mortality when we excluded the age factor due to the small number of our age groups. In addition, according to the study we base it on [14], we need to study with more extensive patient data for the rationality of the seventh day NRBC as the peak value. Further research combining ferritin, fibrinogen, d-dimer, procalcitonin, cardiac troponins, and different inflammatory cytokines and NRBC levels would be more effective in predicting death in these individuals.

## Conclusion

In conclusion, we believe that NRBC elevation (>0.01) is significant in predicting mortality in ICU hospitalized patients due to COVID-19. Worse, a high mortality rate is expected, especially with NRBC values of >0.03. Therefore, NRBC monitoring and intervention at high values are life-saving in COVID-19 ICU patients. However, the presence of chronic diseases and the age of >65 years in addition to these patients increases the probability of mortality. Therefore, we think systemic inflammation and hypoxia, similar to ARDS patients, are the main factors that increase mortality in COVID-19 ICU patients with elevated NRBC levels.

## Dodatak

### Acknowledgments

None.

### Funding

None.

### Data availability

The data is available when requested.

### Conflict of interest statement

All the authors declare that they have no conflict of interest in this work.
